# Healthcare utilization and costs in the first two years after heart failure diagnosis: an observational study by phenotype in southwestern Sweden

**DOI:** 10.1186/s12913-026-14020-4

**Published:** 2026-01-13

**Authors:** Jason Davidge, Anders Halling, Björn Agvall

**Affiliations:** 1 Capio Vårdcentral Halmstad, Halmstad, Sweden; 2 Department of Clinical Sciences Malmö, Center for Primary Health Care Research, Lund University, Malmö, Sweden; 3 Department of Research and Development, Region Halland, Halmstad, Sweden

**Keywords:** Heart failure, Heart failure phenotypes, Healthcare utilization, Healthcare economics, Pharmacotherapy

## Abstract

**Aim:**

Assess healthcare utilization and direct costs for heart failure (HF) during the first two years post-diagnosis across HF subgroups.

**Methods:**

This retrospective population-based study included patients with HF aged 40–90 years in Region Halland. HF subgroups were defined based on echocardiographic ejection fraction, including HF with reduced ejection fraction (HFrEF), mildly reduced ejection fraction (HFmrEF), preserved ejection fraction (HFpEF), and no defined phenotype (HF-NDP). NT-proBNP and comorbidities were analyzed as clinical features. HF phenotypes were identified via algorithmic electronic medical records analysis. Data included primary and hospital care (inpatient and outpatient) healthcare utilization and costs during a two-year post-diagnosis during 2015–2017. Utilization covered visits to physicians, nurses, paramedical staff, and inpatient days. Costs were estimated using the Patient Encounter Costing model. HF phenotypes, differentiated by ejection fraction, were identified via algorithmic analysis of electronic medical records. ANOVA with Bonferroni correction compared subgroups; Poisson regression assessed factors associated with longer hospital stays.

**Results:**

A total of 1769 patients were included: 472 (27%) with HFrEF, 318 (18%) HFmrEF, 505 (28%) HFpEF, and 474 (27%) HF-NDP. Hospitalizations represented the largest cost component, accounting for 68% of first-year expenditures. Average total costs per patient were €15,771 in year one and €7,459 in year two. Subgroup-specific costs declined over time: HFrEF (€18,682 to €7,083), HFpEF (€17,052 to €9,289), HFmrEF (€16,513 to €7,946), and HF-NDP (€11,009 to €5,556). HFpEF patients incurred the highest costs in the second year, indicating a sustained burden. Higher NT-proBNP levels and cardiovascular comorbidities were associated with longer lengths of stay (LoS) in both years. In year one, HFrEF served as reference, while HFmrEF had IRR 0.89 (95% CI:0.88–0.93), HFpEF IRR 0.98 (95% CI:0.94–1.01), and HF-NDP IRR 0.75 (95% CI:0.72–0.79). By year two, LoS risk increased for all other subgroups compared to HFrEF, most notably HFpEF (IRR 1.81; 95% CI:1.70–1.94; *p* < 0.001).

**Conclusions:**

Hospitalizations drove first-year costs, especially for HFrEF. Costs declined in year two for all subgroups, but HFpEF remained highest. Continuous care should target HFpEF and other high-risk phenotypes to reduce long-term burden.

**Supplementary Information:**

The online version contains supplementary material available at 10.1186/s12913-026-14020-4.

## Background

Heart failure (HF) is a chronic condition with an estimated prevalence of 2% that increases significantly with age [1–5]. HF is a severe condition with a high mortality that requires adequate management HF in terms of diagnostics and pharmacotherapy to reduce morbidity, mortality, and to improve quality of life [6]. It has been documented that HF patients use a large proportion of total health care resources and thus place a high financial burden on healthcare systems worldwide [4, 7–11]. Previous studies have indicated that about 20% of patients experience readmission within 30 days, and roughly 33% have a cardiovascular-related hospitalization within 100 days [11–13]. It is therefore understood that hospital care accounts for the greatest share of costs, representing approximately 75–80% [4, 7–9]. A previous review has shown wide variation in estimates of costs outside hospital care for heart failure, including outpatient visits, primary care, diagnostics, and pharmacotherapy, highlighting uncertainty in the overall economic burden beyond hospitalization [14]. A study conducted in Sweden revealed that healthcare utilization and costs related to HF were particularly high, especially during the first year after diagnosis. These costs were primarily driven by high hospitalization rates, with all-cause secondary care expenses reaching €12890 per patient in the first year [10].

HF is generally determined by measuring left ventricular ejection fraction (EF) through echocardiography [6]. Based on the EF, HF patients are further subdivided into one of three phenotypes. Guidelines from 2016 available during the study period defined these phenotypes as: HF with reduced ejection fraction (HFrEF), in which the EF < 40%; HF with mildly reduced EF (HFmrEF) in which the EF is between 40 and 49%; and HF with preserved EF (HFpEF), in which the EF is ≥50% [6, 15]. According to the European Society of Cardiology (ESC) Long-Term Registry, in an outpatient setting, 60% of patients have HFrEF, 24% have HFmrEF, and 16% have HFpEF [16]. In contrast, a population-based study from Region Halland, Sweden, reported that among 8775 patients with heart failure, 57% had a conclusive echocardiography. Within this subgroup, 20% of the total cohort had HFrEF, 16% had HFmrEF, and 22% had HFpEF, while 43% of all patients had no defined phenotype (HF-NDP) [15]. The characteristics of HF subgroups differ, and their pharmacotherapy documentation varies. While hospital care is known to be the primary driver of healthcare utilization and costs in HF, there is limited understanding of how these patterns differ across HF subgroups and change over time, particularly when primary care is taken into account.

This study aimed to evaluate the utilization and costs of primary and secondary healthcare associated with HF during the first and second years after diagnosis according to HF subgroups.

## Materials and methods

This is a retrospective, population-based study of patients in Region Halland (RH) diagnosed with HF and the subsequent primary and secondary health care costs associated with their care during the two years following diagnosis. RH, located in southwestern Sweden, has an estimated population of 330,000 residents. Within RH, there are three acute care hospitals, 40 inpatient wards, two emergency departments, 30 outpatient specialized clinics and 48 primary care facilities.

Patient data were collected via the Regional Healthcare Information Platform (RHIP), a platform containing complete and comprehensive data from electronic healthcare records for residents of RH, including both hospital care and public/private primary healthcare facilities [17]. RHIP contains all prescribed medications, clinical investigation results (i.e., laboratory assessments, radiological examinations) and care delivery resources.

### Study population

All patients residing in RH aged 40–90 years, where a HF diagnosis according to ICD-10 (Appendix – Table 1) was documented in the electronic medical record for the first time during the period 2015–2017 were included. The study period (January 1, 2015, to December 31, 2017) was selected to ensure a minimum of two years of follow-up for all patients, allowing for comprehensive analysis of healthcare utilization and costs during the early post-diagnosis period. Eligibility for the study required patients to have two HF diagnoses, with the second, confirmatory diagnosis occurring no less than 30 days following the initial diagnosis. This criterion was applied to confirm diagnostic accuracy and exclude transient or misclassified cases. It also reduced the likelihood of including patients with severe illness and early mortality, which could bias estimates of healthcare utilization and costs. Only care provided within RH was registered and excluded out-of-region hospitalizations and health care visits. In total, 1769 patients fulfilled the inclusion criteria and were further subdivided based on ejection fraction values as determined by echocardiography.

The patients were categorized based on the EF value measured closest to the initial diagnosis, but not exceeding one year before or after diagnosis. HF subgroups were defined based on echocardiographic assessment of left ventricular EF. Echocardiography within ±365 days of the index date was applicable and formed the base for assessing heart failure phenotype; echocardiograms dated > 365 days before or after index were considered noncontemporaneous and were not used for phenotype assignment, with such patients classified as HFNDP [15]. Patients with reduced EF < 40% were classified as having HFrEF. Those with mildly reduced ejection fraction 40–49% were categorized as HFmrEF, and patients with preserved EF ≥50% were classified as HFpEF. Cases in which echocardiographic data were unavailable or recorded more than one year after the index date were grouped as HF-NDP (15). Patients without echocardiographic data were categorized as HF-NDP. NT-proBNP levels and cardiovascular comorbidities were analyzed separately as clinical characteristics but were not used to define subgroup classification. Data regarding sex, age, blood pressure, heart rate, and comorbidities, as classified by ICD-10 (Appendix – Table 1), were collected at the time of diagnosis (index). Comorbidities were selected based on their relevance to heart failure and impact on healthcare utilization. While conditions such as cancer were present, they were typically inactive or treated and had limited influence on HF-specific care, as shown in previous studies [4]. The healthcare visits in the first and second years following the initial diagnosis were recorded. Healthcare visits were categorized into hospital care, which encompassed visits to the emergency department, inpatient and outpatient hospital care, and primary health care. Hospitalizations were included if the primary or secondary diagnosis was coded under ICD-10 Chapter I (cardiovascular diseases). Admissions for psychiatric, gynecological, and other non-cardiovascular conditions were excluded to focus on hospitalizations relevant to heart failure and related comorbidities.

Data were collected regarding kidney function, as determined by the estimated glomerular filtration rate (eGFR), as well as for the cardiac biomarker N-terminal-pro hormone brain natriuretic peptide (NT-proBNP) [18–20]. NT-proBNP values obtained within three months of diagnosis were included to reflect routine clinical practice and capture the early disease phase. A three-month interval was selected to ensure adequate biomarker assessment [19, 20].

Data regarding healthcare costs were retrieved from the RHIP as determined by the patient encounter costing (PEC) model [21, 22]. Using PEC, costs were calculated based on unit costs for resources and quantities used, as well as separately billable procedures and attributable costs, including medications, radiology exams, and laboratory tests. This method has been employed in previous studies to account for costs related to HF and chronic kidney disease [4]. Costs for services that were not internally priced, such as inpatient ward utilization and physician services, were calculated based on total expenditures for that resource (e.g., an inpatient ward, outpatient clinic, ED or primary care facility) divided by the total number of patient encounters or care episodes delivered of that ward or clinic. This costing method may generalize individual resource use, but it enables consistent estimation across large cohorts and has been validated in previous Region Halland studies. Its impact on subgroup comparisons is considered minimal due to uniform application. For inpatient care, the unit of analysis was hospital bed days. For outpatient and primary care, it was clinic visits. The cost of a particular encounter was calculated as the amount of resource used (e.g., bed days in hospital) multiplied by the unit cost for that resource, plus all separate cost procedures or other attributable costs. The cost of all encounters was added together to obtain the total cost accrued for each patient during the study year. This allocation approach assumes average resource use per encounter; however, the PEC model applies time-driven activity-based costing principles, using resource capacity (e.g., bed-days, staff time) to reduce bias from capacity fluctuations. Costs were originally retrieved in Swedish kronor (SEK) and converted to euros (EUR) using the 2017 average exchange rate of 9.64 SEK per EUR.

### Statistics

Descriptive statistics were used to describe the study cohort. Categorical data were analyzed using Pearson’s Chi-2 test, and Mann–Whitney U-test was used when comparing continuous data. Kruskal-Wallis tests were applied for comparison of more than two groups.

The study cohort was grouped according to gender and age. Patients were categorized based on age as ≤ 75 and > 75 years. Age and gender were analyzed with Pearson’s Chi-2 test. The NT-proBNP values were divided into three groups to determine the likeliness that they were associated with HF. Those with normal NT-proBNP levels were defined as “HF unlikely” and elevated levels were defined based on patient age as either “grey zone” or “HF likely”, as shown in detail in Appendix - Table 2 [19, 20]. Kidney function was considered normal with an eGFR ≥60 ml/min, reduced with eGFR 30–59 ml/min or impaired with eGFR < 30 ml/min [18]. For continuous NT-proBNP values, which showed a markedly skewed distribution, data were presented as median and interquartile range, and the Kruskal Wallis test was applied to compare distributions across heart failure phenotypes. Kruskal-Wallis test was used to analyze the groups for age, eGFR and NT-proBNP, when comparing the HF-phenotypes.

Healthcare encounters were recorded for primary- and secondary care for each HF subgroup based on data available in RHIP. Differences in mean healthcare utilization between the HF subgroups were analyzed using one-way ANOVA. As the data regarding healthcare costing showed a right-skewed distribution, the data was normalized using logarithmic transformation to enable parametric statistical analysis. Differences in mean cost values between HF subgroups were analyzed using one-way ANOVA. When the overall analysis of variance showed significance, a post hoc test with Bonferroni correction was applied to determine which of the HF subgroups differed significantly from the others for the healthcare resource in question. As the cost data were normalized using logarithmic transformation, the inverse function antilog was applied to the mean differences to determine the cost difference between HF subgroups in percentage.

A Poisson regression model was used to analyze the number of hospital care days during the first- and second-years post-diagnosis. The model included adjustments for relevant covariates: age, HF subgroup, comorbidities, kidney function, and categorized NT-proBNP levels. Patients who died during the first year were excluded from the second-year analysis of healthcare utilization and costs. Poisson regression models were applied separately each year, using the respective cohort of patients alive during that period. Prior to analysis, overdispersion was assessed by comparing the Pearson chi-square statistic to its degrees of freedom, and no significant overdispersion was detected, supporting the appropriateness of the Poisson model. In addition to the main Poisson model for hospital days, multivariable Poisson regressions were performed for other utilization measures (primary care, outpatient, emergency visits), adjusting for age, sex, comorbidities, kidney function, and NTproBNP. A p-value < 0.05 was considered significant. Data was analyzed using IBM SPSS Statistics 29, Armonk, New York, USA.

## Results

A total of 1769 patients were included in the study, and the distribution of HF subgroups was 472 (27%) HFrEF, 318 (18%) HFmrEF, 505 (28%) HFpEF, and 474 (27%) HF-NDP. The cohort included 987 (56%) men and 782 (44%) women. Among HFNDP, 145 patients had a normal echocardiogram (EF ≥ 50%) dated > 365 days before index. Among those with HFrEF and HFmrEF, 67 and 63% were men, respectively, with a higher prevalence of arteriosclerotic cardiovascular disease (ASCVD). Men had a higher prevalence in HFrEF and HFmrEF and were slightly more prevalent in HFpEF, whereas women were more prevalent in HF-NDP. HFpEF showed higher prevalence rates of hypertension, atrial fibrillation, and COPD. Detailed results on gender, age, comorbidities, kidney function, and NT-proBNP levels across HF subgroups are summarized in Table 1.

**Table 1:** Distribution and clinical characteristics of the study population at diagnosis in total and by HF-subgroups

	Total					
		HF confirmed by echocardiography			No echo	
		**HFrEF**	**HFmrEF**	**HFpEF**	**HF-NDP**	**p-value**
Total, n (%)	1769 (100)	472 (27)	318 (18)	505 (28)	474 (27)	< 0.001^1^
Women, n (%)	782 (44)	158 (33)	118 (37)	245 (49)	261 (55)	< 0.001^1^
Men, n (%)	987 (56)	314 (67)	200 (63)	260 (51)	213 (45)	
Age, mean (SD)	78.4 (11.5)	73.4 (11.8)	75.6 (10.6)	79.4 (10.1)	84.1 (10.2)	< 0.001^2^
Age ≥ 75 years (%)	1204 (68)	239 (51)	180 (57)	373 (74)	412 (87)	< 0.001^1^
*Comorbidities*						
Hypertension, n (%)	1214 (69)	258 (55)	210 (66)	400 (79)	346 (73)	< 0.001^1^
ASCVD, n (%)	858 (49)	252 (53)	177 (56)	224 (44)	205 (43)	< 0.001^1^
Atrial fibrillation, n (%)	889 (50)	184 (39)	157 (49)	303 (60)	245 (52)	< 0.001^1^
Diabetes mellitus, n (%)	396 (22)	108 (23)	72 (23)	123 (24)	93 (20)	0.35^1^
COPD, n (%)	261 (15)	54 (11)	42 (13)	88 (17)	77 (16)	0.03^1^
*Kidney function*						
eGFR, mean (SD)	55.5 (18.2)	59.2 (19.2)	56.8 (18.8)	54.4 (17.3)	52.1 (17.1)	< 0.001^2^
eGFR >60 ml/min, n (%)	778 (44)	250 (53)	158 (50)	205 (41)	165 (35)	< 0.001^1^
eGFR 59–30 ml/min, n (%)	829 (47)	182 (39)	126 (40)	257 (51)	264 (56)	
eGFR < 30 ml/min, n (%)	151 (9)	38 (8)	30 (10)	43 (9)	40 (9)	
*Natriuretic peptide*						
NT-proBNP ng/l, median (IQR)	3349 (6333)	4794 (9675)	2875 (5520)	3100 (5014)	2451 (5207)	< 0.001^2^
HF Unlikely^3^, n (%)	70 (4)	8 (2)	12 (4)	23 (5))	27 6)	< 0.001^1^
“Grey Zone”^4^, n (%)	293 (47)	37 (50)	61 (51)	92 (42)	103 (22)	
HF Likely^5^, n (%)	1215 (52)	412 (50)	226 (48)	352 (58)	225 (47)	
NT-proBNP missing, n (%)	191 (11)	15 (3)	19 (6)	38 (8)	119 (25)	

Note: *n* = numbers; SD = standard deviation; IQR = HFrEF = Heart failure (HF) with reduced ejection fraction (EF); HFmrEF = HF with mildly reduced EF; HFpEF = HF with preserved EF; HF-NDP = heart failure with no defined phenotype; ASCVD = atherosclerotic cardiovascular diseases including ischemic heart disease, peripheral artery disease, myocardial infarction and stroke; COPD = chronic obstructive pulmonary disease; eGFR = estimated Glomerular filtration rate; NT-proBNP = N-terminal pro b-type natriuretic peptide

^1^Pearson Chi-2 test

^2^Kruskal-Wallis test

^3^HF Unlikely = NT-proBNP < 300 pg/mL for all ages

^4^“Grey Zone” = NT-proBNP 300–450 pg/mL age < 50; 300–900 pg/mL age 50–75; 300–1800 pg/mL age > 75

^5^HF Likely= > 450 pg/mL age < 50; > 900 pg/mL age 50–75; > 1800 pg/mL age > 75

P-values refer to overall comparisons across HF subgroups (HFrEF, HFmrEF, HFpEF, HF-NDP). Pearson’s Chi-squared test was used for categorical variables and Kruskal–Wallis test for continuous variables. For multi-category variables (e.g., eGFR), the p-value reflects the overall distribution comparison across all categories

Of the 1769 patients enrolled, 475 (27%) died during the two-year follow-up period, with 282 (16%) of these deaths occurring within the first year.

The distribution of per patient healthcare encounters and costs during the first and second year after diagnosis are displayed in Table 2 for the total cohort and for each HF-subgroup. The most expensive subgroup in the first year was HFrEF patients with a mean total cost per patient of €18682. In the second year, it was the HFpEF subgroup with a mean total cost per patient of €9289.

**Table 2:** Distribution of per patient healthcare utilization and costs within the first two years after HF diagnosis based on subgroup

	All Patients	HFrEF	HFmrEF	HFpEF		HF-NDP	p-value
	Mean (SD)	Mean (SD)	Mean (SD)	Mean (SD)		Mean (SD)	
**Healthcare utilization**							
**First year** (*n* = 1769)							
Inpatient care							
*bed* days *(LoS)*	14.5 (7.3)	16.7 (8.0)	15.1 (7.2)	15.7 (7.1)		10.7 (6.3)	< 0.001
*admissions*	2.0 (1.8)	2.1 (1.6)	2.1 (2.0)	2.2 (2.0)		1.7 (1.6)	< 0.001
Hospital outpatient care							
*ED visits*	2.2 (2.3)	2.2 (2.1)	2.3 (2.9)	2.4 (2.4)		1.9 (1.9)	0.002
*OPC visits*	9.3 (14.8)	13.8 (14.3)	11.2 (16.6)	9.0 (13.1)		4.0 (13.9)	< 0.001
Primary health care							
*PC visits*	20.2 (14.8)	19.1 (17.6)	20.1 (16.2)		23.4 (17.5)	18.0 (17.6)	< 0.001
**Second year** (*n* = 1487)							
Inpatient care							
*bed days (LoS)*	5.0 (6.3)	3.8 (5.4)	5.0 (7.1)		6.5 (6.5)	4.2 (6.0)	0.001
*admissions*	0.8 (1.4)	0.7 (1.3)	0.7 (1.4)		1.0 (1.6)	0.7 (1.3)	< 0.001
Hospital outpatient care							
*ED*	0.9 (1.7)	0.8 (1.7)	0.9 (1.6)		1.2 (1.9)	0.9 (1.5)	0.003
*Outpatient care visits*	5.4 (13.9)	7.2 (15.5)	5.1 (11.5)		6.0 (14.9)	3.3 (12.4)	< 0.001
Primary care							
*PC visits*	14.0 (16.8)	13.7 (16.6)	13.0 (14.5)		16.7 (18.3)	12.3 (16.4)	< 0.001
**Healthcare costs**							
**First year** (*n* = 1769)							
Inpatient care							
*bed days (LoS)*	10834	13325	10613		11834	7435	< 0.001
Hospital outpatient care							
*Emergency department visits*	778	763	839		891	632	0.04
*Outpatient care visits*	1875	2405	2379		1835	1050	< 0.001
Primary care							
*PC visits*	978	996	947		1044	912	0.01
Medications	1306	1193	1736		1448	980	< 0.001
**Costs for first year**	**15771**	**18682**	**16513**		**17052**	**11009**	< 0.001
**Second year** (*n* = 1487)							
Inpatient care							
*bed days (LoS)*	3995	3420	4330		5115	2895	0.15
Hospital outpatient care							
*Emergency department visits*	354	291	383		453	272	0.29
*Outpatient care visits*	1487	1738	1295		1961	790	< 0.001
Primary care							
*PC visits*	501	546	467		525	418	0.63
Medications	1121	1088	1471		1236	1180	< 0.001
**Costs for second year**	**7459**	**7083**	**7946**		**9289**	**5556**	< 0.001

Note: LoS = Length of stay; ED = Emergency department; PC = Primary care facilities. P-values refer to overall comparisons across HF subgroups (HFrEF, HFmrEF, HFpEF, HF-NDP) using one-way ANOVA. Log-transformed data were used where appropriate to account for skewness. Post hoc tests with Bonferroni correction were applied when overall significance was observed

**Fig. 1:**
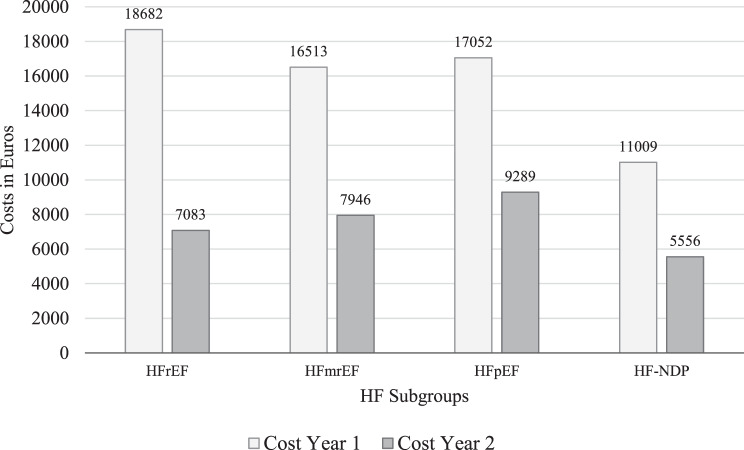
The costs for each HF-subgroup representing the first and second year after the onset of HF

The main cost driver for all subgroups was hospitalization/inpatient care, accounting for 68% of the total cost of patient care in the first year alone. The highest average cost per patient in the first year was €18682 for the HFrEF subgroup, which decreased to €7083 by the second year. Similar cost reductions were observed across all HF subgroups, although the smallest decreases were seen in patients with HFpEF and HF-NDP. HFpEF patients had the highest cost in the second year at €9289. The distribution of costs for each HF-subgroup for the first and second year after the onset of HF is illustrated in Fig. 1.

A Poisson distribution was used to identify factors increasing the probability of longer hospital stays is presented in Table 3. In the first year, the HFrEF subgroup showed that patients with ASCVD, COPD, diabetes, higher NT-proBNP levels, and eGFR < 30 ml/min were associated with longer hospital stays, after adjustment for heart failure subgroup and other covariates. In the second year, the HFpEF subgroup had the highest incidence rate ratio of 1.81 (95% CI: 1.70–1.94). The presence of ASCVD, COPD, and higher NT-proBNP levels were associated with extended hospital stays in both the first and the second year. Adjusted incidence rate ratios for primary care, hospital outpatient, and emergency visits during both years are presented in Appendix Table 3.

**Table 3:** Poisson regression analysis showing factors associated with longer hospital stays in the first and second year after HF diagnosis

		First year				Second year		
	IRR	95% Wald CI		p-value	IRR	95% Wald CI		p-value
		**Lower**	**Upper**			**Lower**	**Upper**	
*Age*	1.00	0.99	1.00	0.17	0.998	0.996	0.999	0.01
*Sex*								
Female	1.00				1.00			
Male	0.86	0.84	0.89	< 0.001	1.03	0.98	1.09	0.20
*HF Subgroup*								
HFrEF	1.00				1.00			
HFmrEF	0.89	0.88	0.93	< 0.001	1.16	1.01	1.26	< 0.001
HFpEF	0.98	0.94	1.01	0.20	1.81	1.70	1.94	< 0.001
HF-NDP	0.75	0.72	0.79	< 0.001	1.13	1.04	1.22	< 0.001
*Comorbidities*								
ASCVD	1.34	1.30	1.38	< 0.001	1.22	1.16	1.28	< 0.001
COPD	1.34	1.30	1.39	< 0.001	1.31	1.23	1.39	< 0.001
DM	1.14	1.10	1.12	< 0.001	1.35	1.28	1.43	< 0.001
AF	0.98	0.95	1.01	0.10	1.01	0.96	1.06	0.71
*eGFR*								
≥60 ml/min	1.00				1.00			
30–59 ml/min	0.91	0.88	0.94	< 0.001	0.91	0.86	0.96	< 0.001
< 30 ml/min	1.33	1.27	1.39	< 0.001	0.92	0.85	0.99	0.23
*NT-proBNP*								
HF Unlikely^1^	1.00				1.00			
Grey Zone^2^	1.43	1.29	1.58	< 0.001	1.62	1.38	1.90	< 0.001
HF Likely^3^	2.28	2.07	2.51	< 0.001	1.90	1.63	2.22	< 0.001

Note: IRR = Incidence rate ratio. HFrEF = Heart failure (HF) with reduced ejection fraction (EF); HFmrEF = HF with mildly reduced EF; HFpEF = HF with preserved EF; HF-NDP = heart failure with no defined phenotype; ASCVD = atherosclerotic cardiovascular diseases including ischemic heart disease, peripheral artery disease, myocardial infarction and stroke; COPD = chronic obstructive pulmonary disease; eGFR = estimated Glomerular filtration rate; NT-proBNP = N-terminal pro b-type natriuretic peptide

^1^HF Unlikely = NT-proBNP < 300 pg/mL for all ages

^2^“Grey Zone” = NT-proBNP 300–450 pg/mL age < 50; 300–900 pg/mL age 50–75; 300–1800 pg/mL age > 75

^3^HF Likely= > 450 pg/mL age < 50; > 900 pg/mL age 50–75; > 1800 pg/mL age > 75

## Discussion

In this retrospective observational study of HF patients, healthcare utilization and costs were found to be highest in the first year, especially among those with HFrEF. The primary cost driver during both years was due to hospitalization. By the second year, there was a reduction in healthcare utilization and costs across all HF subgroups, with the largest decrease observed in the HFrEF group. This decrease was not as evident in the HFpEF group, which emerged as the costliest subgroup in the second year. This may reflect persistent comorbidity burden in the HFpEF group and limited treatment options, contributing to continued healthcare needs beyond the initial stabilization phase [6]. The factors associated with the number of hospital days varied by HF subgroup, with HFrEF being the predominant factor in the first year and HFpEF in the second year. Across both years, ASCVD, COPD, diabetes mellitus, impaired kidney function, and high NT-proBNP levels were associated with an increased number of hospital days.

During the first year following HF diagnosis, patients exhibited substantial healthcare utilization, primarily through inpatient hospital care. On average, each patient spent 14.5 days in the hospital during this initial year, decreasing distinctly to 5.0 days in the second year. The demand for hospital care initially was pronounced, and other studies have similarly highlighted a heightened risk of readmission following discharge from HF-related hospitalizations [11–13]. This pattern was most pronounced in patients with HFrEF, who averaged 16.7 hospital days in the first year, decreasing to 3.8 days in the second year. Previous research indicates an average of 6.6 hospital bed-days per year in a prevalent HF cohort [4, 7, 8]. Comparative data for the first and second year after diagnosis in incident HF cohorts are limited, highlighting the importance of these findings. In terms of outpatient care, patients with HF made an average of 20.2 visits to primary care in the first year, which decreased to 14.0 visits in the second year. During the first year, patients with HFrEF predominantly visited outpatient hospital settings, while patients with HFpEF primarily attended visits in primary care settings. HFrEF and HFpEF showed similar utilization in year one, likely due to shared early-phase management needs. HFmrEF differed more, reflecting its transitional nature. By year two, most patients stabilized, and HFpEF accounted for a relatively higher share of encounters, suggesting persistent complexity.

The costs for HF patients amounted to €15771 in the first year after diagnosis, decreasing significantly to €7459 in the second year. Inpatient care emerged as the main cost driver in this patient category, a finding similar to other studies [4, 7–11]. Earlier studies reported annual costs up to €9790, in contrast to our findings [4]. A study in Sweden highlighted particularly high healthcare utilization and costs associated with HF, especially in the initial year post-diagnosis [10]. These costs were predominantly driven by frequent hospitalizations, with total secondary care expenses reaching €12890 per patient in the first year. The lower cost observed in this study compared to ours can be attributed to its focus solely on secondary care, without accounting for primary care expenditures.

Given that inpatient care is the primary cost driver and hospitalization is most critical in the first year, it is understandable that costs peak during this period, especially for patients with HFrEF. A prior study highlighted significant resource utilization among HFpEF patients, largely driven by hospitalizations [23]. Specifically, HF-related admissions accounted for 50% and cardiovascular-related admissions for 32% of total resource utilization. Moreover, findings indicated that 60% of HFpEF patients experienced at least one hospitalization during follow-up. These hospitalizations, particularly those related to HF, constitute a substantial portion of the overall healthcare burden for HFpEF patients in Sweden. Our study also reveals that patients with HFpEF incur significant costs, which do not decrease to the same extent in the second year. This indicates that HFpEF stands out as the HF subgroup with the highest costs in the second year. As a result, patients with HFpEF face prolonged and more extensive healthcare expenditures, which often involve primary care to a greater extent compared to patients with HFrEF. Managing patients with HFpEF has been challenging, but in recent years, sodium–glucose co-transporter 2 inhibitors have been recommended along with treatments addressing the underlying causes, cardiovascular issues, and other health conditions to mitigate hospital admissions [24]. Implementing comprehensive management strategies in this context could potentially decrease hospital admissions, a responsibility that primarily lies within primary care settings.

The comorbidities and features concurrent to the highest number of hospital bed days were ASCVD, COPD, diabetes, and elevated NT-proBNP levels, regardless of the year. The consistent association for the eGFR 30–59 group across both years may reflect clinical factors such as closer monitoring and treatment adjustments in patients with moderate kidney impairment, which could influence hospitalization duration. This interpretation is not directly confirmed by the present data. Previous studies have reported similar associations between morbidity and these comorbidities, but the current study finds that this association persist over time [10]. These factors consistently influence the need for inpatient care over time and should be considered when assessing patients with HF.

Comorbidities associated with HF certainly contribute, as previous studies have suggested that as much as 70% of readmissions were attributed to non-HF-related causes [25]. However, the impact varies across HF subgroups, suggesting that the approach to planned follow-up may differ, potentially affecting costs [13, 26, 27].

### Strengths and limitations

A major strength of this study is the robustness of the cohort. As opposed to HF registry studies, the present study examined an unselected, population-based cohort, which shows a more even distribution with greater homogeneity. In addition, compared to previous studies that examined all HF patients with a healthcare encounter, the present study cohort required two HF diagnoses, with the second recorded at least 30 days after the initial diagnosis. Since mortality is highest shortly after the initial diagnosis, patients who received a second heart failure diagnosis within one month were excluded. These individuals were considered either to be severely ill, with limited contribution to long-term healthcare costs, or potentially misdiagnosed, which could introduce bias in cost estimations.

However, some limitations should be acknowledged. First, attributing costs exclusively to HF is challenging because patients often have multiple comorbidities. Second, the findings are associative rather than causal, given the observational design. Third, the analysis focused on clinics managing HF and excluded non-medicine clinics such as psychiatry. Additionally, only direct healthcare costs were analyzed; indirect costs were not included, which limits the comprehensiveness of the economic evaluation.

The study cohort included patients residing in Region Halland and listed for care within the region during the study period. Only healthcare encounters and costs recorded within Region Halland were analyzed. While occasional care outside the region may have occurred, such events were registered but not detailed and are considered minimal. The study design supports the assumption that most care was delivered within the regional system, limiting the risk of selection bias.

## Conclusion

The study found substantially higher healthcare utilization and costs in the first year compared to the second year, driven mainly by inpatient care. Healthcare encounters and subsequent costs did reduce considerably in the second year. Hospital admissions were the main cost driver amongst all HF subgroups. Impaired kidney function, elevated NT-proBNP levels, ASCVD, COPD, and diabetes associate with a higher need for hospitalization in both the first and second years. In the first year, patients with HFrEF require the most healthcare resources, but by the second year, those with HFpEF become the main subgroup necessitating healthcare resources. These findings imply that interventions should involve personalized care plans tailored to HF subtypes and associated comorbidities to enhance patient outcomes and manage costs effectively.

## Electronic supplementary material

Below is the link to the electronic supplementary material.

Supplementary Material 1

## Data Availability

The datasets generated and/or analyzed during the current study are not publicly available due to privacy regulations but are available from the corresponding author on reasonable request and pending approval by Region Halland.
