# Supplementary material for: Healthcare utilization and costs in the first two years after heart failure diagnosis: an observational study by phenotype in southwestern Sweden
Source: BMC Health Serv Res. 2026 Jan 13;26:148. doi: 10.1186/s12913-026-14020-4 (PMC12849546; doi:10.1186/s12913-026-14020-4)
Supplement: Supplementary file 1 — Supplementary Material 1 [file 12913_2026_14020_MOESM1_ESM.docx]

| Appendix-Table I. ICD 10 codes for the comorbidities included in the study. | |
| --- | --- |
|  |  |
| **Disease** | **ICD-10 codes** |
|  |  |
| Heart failure | I110, I420, I423 - I432, I438, I500 - I501, I509 |
| Hypertension | I10 - I15 |
| Ischemic heart disease | I20 - I25 |
| Cerebrovascular insult | I60 – i69 |
| Atrial fibrillation | I48 |
| Diabetes mellitus | E10 - E14 |
| Chronic obstructive pulmonary disease | J40 - J44 |
| Chronic kidney disease | N17 -N19 |
| Acute myocardial infarction | I21 - I22 |
| Peripheral artery disease | I739 |
| Valvular heart disease | I06, I34-I35, Q230, Q231, Q233 |

| Appendix Table II. Recommended N-terminal pro brain natriuretic peptide (NT-proBNP) cut-offs for heart failure diagnosis. | | | | |
| --- | --- | --- | --- | --- |
|  | | | | |
| **Cut-off levels (ng/L)** | | | |  |
|  |  |  |  |  |
|  | **Age < 50 years** | **Age 50 - 75 years** | **Age > 75 years** |  |
|  |  |  |  |  |
| HF unlikely | < 300 |  |  |  |
|  |  |  |  |  |
| "Grey Zone" | 300 - 450 | 300 - 900 | 300 - 1800 |  |
|  |  |  |  |  |
| HF likely | >450 | > 900 | > 1800 |  |
|  |  |  |  |  |
| HF = heart failure; NT-proBNP = N-terminal pro brain natriuretic peptide | | | | |

Appendix Table 3. Adjusted IRRs for primary care, outpatient, and emergency visits in first- and second-year post‑HF diagnosis from Poisson models.

|  | **First year** | | | | **Second year** | | | |
| --- | --- | --- | --- | --- | --- | --- | --- | --- |
|  |  | **95% CI** | |  |  | **95% CI** | |  |
|  | **IRR** | **Lower** | **Upper** | **p-value** | **IRR** | **Lower** | **Upper** | **p-value** |
| *Primary care* |  |  |  |  |  |  |  |  |
| HFrEF | 1 |  |  |  | 1 |  |  |  |
| HFmrEF | 1.01 | 0.98 | 1.04 | 0.63 | 0.91 | 0.87 | 0.95 | <0.001 |
| HFpEF | 1.13 | 1.09 | 1.16 | <0.001 | 1.12 | 1.08 | 1.16 | <0.001 |
| HFNDP | 0.95 | 0.91 | 0.98 | 0.002 | 0.94 | 0.9 | 0.98 | 0.008 |
| Age | 1 | 1.00 | 1.00 | 0.87 | 1 | 1.00 | 1.00 | 0.19 |
| Male | 1 |  |  |  | 1 |  |  |  |
| Women | 0.89 | 0.87 | 0.91 | <0.001 | 0.87 | 0.85 | 0.89 | <0.001 |
| ASCVD | 1.01 | 0.99 | 1.03 | 0.46 | 0.97 | 0.94 | 0.99 | 0.03 |
| Atrial fibrillation | 1.35 | 1.32 | 1.38 | <0.001 | 1.44 | 1.4 | 1.48 | <0.001 |
| Diabetes mellitus | 1.38 | 1.35 | 1.42 | <0.001 | 1.52 | 1.48 | 1.57 | <0.001 |
| COPD | 1.13 | 1.09 | 1.16 | <0.001 | 1.24 | 1.2 | 1.29 | <0.001 |
| CKD stage 1-2 | 1 |  |  |  | 1 |  |  |  |
| CKD stage 3 | 1.03 | 1.001 | 1.05 | 0.05 | 0.98 | 0.95 | 1.01 | 0.13 |
| CKD stage 4-5 | 1.17 | 1.13 | 1.22 | <0.001 | 1.22 | 1.16 | 1.29 | <0.001 |
| HF unlikely | 1 |  |  |  | 1 |  |  |  |
| HF Greyzone | 1.13 | 1.06 | 1.2 | <0.001 | 1.54 | 1.42 | 1.67 | <0.001 |
| HF likely | 0.98 | 0.92 | 1.04 | 0.55 | 1.29 | 1.19 | 1.39 | <0.001 |
| *Outpatient hospital care* | |  |  |  |  |  |  |  |
| HFrEF | 1 |  |  |  | 1 |  |  |  |
| HFmrEF | 0.88 | 0.85 | 0.92 | <0.001 | 0.74 | 0.7 | 0.79 | <0.001 |
| HFpEF | 0.84 | 0.8 | 0.87 | <0.001 | 1.07 | 1.01 | 1.13 | 0.02 |
| HFNDP | 0.36 | 0.34 | 0.39 | <0.001 | 0.61 | 0.56 | 0.66 | <0.001 |
| Age | 0.97 | 0.97 | 0.97 | <0.001 | 0.97 | 0.97 | 0.97 | <0.001 |
| Male | 1 |  |  |  | 1 |  |  |  |
| Women | 0.86 | 0.83 | 0.89 | <0.001 | 0.77 | 0.73 | 0.8 | <0.001 |
| ASCVD | 0.99 | 0.96 | 1.03 | 0.63 | 0.98 | 0.94 | 1.03 | 0.48 |
| Atrial fibrillation | 1.04 | 1.01 | 1.08 | 0.02 | 1.02 | 0.97 | 1.07 | 0.42 |
| Diabetes mellitus | 1.1 | 1.06 | 1.14 | <0.001 | 1.25 | 1.19 | 1.31 | <0.001 |
| COPD | 0.93 | 0.88 | 0.97 | 0.002 | 0.97 | 0.91 | 1.03 | 0.32 |
| CKD stage 1-2 | 1 |  |  |  | 1 |  |  |  |
| CKD stage 3 | 1.03 | 0.99 | 1.07 | 0.21 | 1.21 | 1.15 | 1.28 | <0.001 |
| CKD stage 4-5 | 1.59 | 1.5 | 1.68 | <0.001 | 3.16 | 2.95 | 3.39 | <0.001 |
| HF unlikely | 1 |  |  |  | 1 |  |  |  |
| HF Greyzone | 1.29 | 1.17 | 1.43 | <0.001 | 1.68 | 1.46 | 1.93 | <0.001 |
| HF likely | 1.53 | 1.39 | 1.67 | <0.001 | 1.94 | 1.7 | 2.21 | <0.001 |
| *Emergency department* | |  |  |  |  |  |  |  |
| HFrEF | 1 |  |  |  | 1 |  |  |  |
| HFmrEF | 1.01 | 0.91 | 1.11 | 0.87 | 1.04 | 0.89 | 1.22 | 0.64 |
| HFpEF | 1.1 | 1.01 | 1.2 | 0.04 | 1.32 | 1.15 | 1.52 | <0.001 |
| HFNDP | 0.91 | 0.82 | 1.01 | 0.08 | 0.97 | 0.82 | 1.16 | 0.76 |
| Age | 1.01 | 1.00 | 1.01 | 0.003 | 1.01 | 1.00 | 1.02 | <0.001 |
| Male | 1 |  |  |  | 1 |  |  |  |
| Women | 1.04 | 0.97 | 1.11 | 0.26 | 0.97 | 0.88 | 1.08 | 0.62 |
| ASCVD | 1.18 | 1.1 | 1.26 | <0.001 | 1.32 | 1.19 | 1.47 | <0.001 |
| Atrial fibrillation | 1.08 | 1.01 | 1.15 | 0.04 | 1.21 | 1.09 | 1.35 | <0.001 |
| Diabetes mellitus | 1.06 | 0.98 | 1.14 | 0.16 | 1.17 | 1.04 | 1.32 | 0.01 |
| COPD | 1.35 | 1.24 | 1.47 | <0.001 | 1.43 | 1.26 | 1.63 | <0.001 |
| CKD stage 1-2 | 1 |  |  |  | 1 |  |  |  |
| CKD stage 3 | 0.83 | 0.77 | 0.9 | <0.001 | 0.82 | 0.73 | 0.93 | 0.001 |
| CKD stage 4-5 | 1.05 | 0.93 | 1.19 | 0.39 | 0.97 | 0.79 | 1.2 | 0.79 |
| HF unlikely | 1 |  |  |  | 1 |  |  |  |
| HF Greyzone | 1.25 | 1.01 | 1.55 | 0.04 | 1.12 | 0.83 | 1.5 | 0.46 |
| HF likely | 1.47 | 1.2 | 1.8 | <0.001 | 1.19 | 0.9 | 1.58 | 0.22 |

Notes: HFrEF = heart failure with reduced ejection fraction; HFmrEF = heart failure with mildly reduced ejection fraction; HFpEF = heart failure with preserved ejection fraction; HF‑NDP = heart failure with no defined phenotype; ASCVD = atherosclerotic cardiovascular disease; AF = atrial fibrillation; DM = diabetes mellitus; COPD = chronic obstructive pulmonary disease; CKD = chronic kidney disease (stage 1–2: eGFR ≥60 mL/min/1.73m²; stage 3: 30–59; stage 4–5: <30); NT‑proBNP = N‑terminal pro‑B‑type natriuretic peptide; PC = primary care; OPC = outpatient hospital clinic; ED = emergency department; IRR = incidence rate ratio; CI = confidence interval.

Model specification:
Incidence rate ratios (IRR) are from multivariable Poisson regression with log link, adjusted for age, sex, ASCVD, AF, DM, COPD, CKD stage, and NT‑proBNP category. The reference categories are HFrEF (phenotype), men (sex), CKD stage 1–2 (eGFR ≥60), and NT‑proBNP “HF unlikely.” Models included an offset for log (days alive in the respective year) to account for varying exposure time.

.
